# Transcriptomic Analysis Reveals That the Photosynthesis and Carotenoid Metabolism Pathway Is Involved in the Salinity Stress Response in *Brassica rapa* L. ssp. *Pekinensis*

**DOI:** 10.3390/plants14040566

**Published:** 2025-02-13

**Authors:** Jin Jia, Fengshuo Wang, Mengmeng Yuan, Zhiying Wang, Zhe Qin, Xiaoli Zhang, Yutao Shao, Haixia Pei

**Affiliations:** School of Life Science and Technology, Inner Mongolia University of Science & Technology, Baotou 014010, China; wangfengshuozs@163.com (F.W.); y18047233942@163.com (M.Y.); wangzhiying0916@163.com (Z.W.); qinzhe1990@outlook.com (Z.Q.); zxl_6018@imust.edu.cn (X.Z.); syt_234@163.com (Y.S.); phx2003@126.com (H.P.)

**Keywords:** *Brassica rapa* L. ssp. *Pekinensis*, salinity, carotenoid metabolism, RNA-seq, chlorophyll

## Abstract

Salinity stress is a major abiotic factor that adversely affects plant growth and development. This study investigated the physiological and molecular responses of *Brassica rapa* L. ssp. *Pekinensis* to salinity stress by subjecting seedlings to varying concentrations of NaCl. Physiological analysis revealed significant wilting, chlorosis, and a marked reduction in chlorophyll and carotenoid contents in NaCl-treated seedlings, indicating impaired photosynthetic efficiency and oxidative stress mitigation. RNA-seq analysis identified extensive transcriptional reprogramming, with 6693 and 10,280 differentially expressed genes (DEGs) in Z150 and Z300 treatments, respectively, compared to the control group. DEGs were clustered into six expression trends, with sustained up-regulation in Clusters 2 and 6 and down-regulation in Cluster 3. Gene Ontology (GO) enrichment analysis highlighted the involvement of these DEGs in stress responses. Key DEGs encoding heat shock proteins, peroxidases, glutathione S-transferases, and transcription factors were significantly induced under salinity stress, suggesting their roles in stress adaptation. Furthermore, GO and KEGG enrichment analyses revealed significant down-regulation of genes associated with photosynthesis and carbon metabolism, indicating disruption of these critical pathways. Weighted Gene Co-expression Network Analysis (WGCNA) identified hub genes, such as histidine synthase and low-density receptor-like protein, potentially central to salinity stress responses. Additionally, carotenoid metabolism was significantly inhibited, with down-regulation of key genes in the carotenoid biosynthesis pathway. RT-qPCR validation confirmed the reliability of the RNA-seq data. Collectively, these findings provide comprehensive insights into the physiological and molecular mechanisms underlying response of *B. rapa* L. ssp. *Pekinensis* to salinity stress, highlighting potential targets for improving salinity tolerance in crops.

## 1. Introduction

As one of the vegetables with the longest history of cultivation and consumption in China, Chinese cabbage (*Brassica rapa* L. ssp. *Pekinensis*) is rich in vitamins and has high nutritional value, helping to replenish the vitamins needed by the human body and providing antioxidant benefits [1]. The presence of dietary fiber in Chinese cabbage not only assists in digestion but also offers medicinal benefits [2]. Nevertheless, the production of Chinese cabbage in regions like Inner Mongolia is constrained by salinity stress [3].

Salinity stress is one of the most significant abiotic factors limiting plant growth and crop productivity worldwide [4]. As global climate change progresses and agricultural practices intensify, the problem of soil salinization is expected to worsen, threatening food security for a growing world population [5]. The current estimate suggests that over 6% of the total land area on Earth and 20% of irrigated agricultural land are facing salinity issues, and these numbers are forecasted to increase in the years to come [6]. Therefore, it is essential to comprehend how plants react and adjust to salinity stress in order to develop tactics for enhancing crop salt tolerance and sustaining agricultural productivity in areas affected by salt. Salinity stress induces a complex array of physiological, biochemical, and molecular responses in plants [7]. The primary effects of high soil salinity on plants are osmotic stress and ion toxicity [8,9]. Osmotic stress occurs when there is a high concentration of solutes in the soil solution, causing a decrease in the soil water potential that hinders plants from absorbing water effectively from the soil. Consequently, this results in a physiological drought state, even if the soil moisture levels are sufficient [10,11]. Excessive accumulation of Na+ and Cl− ions in plant tissues can lead to ion toxicity, disrupting cellular homeostasis, enzyme function, and various metabolic processes [6].

To cope with these challenges, plants have evolved various adaptive mechanisms at the molecular, cellular, and whole-plant levels [12]. These strategies include ion exclusion, tissue tolerance, and osmotic adjustment [13]. Salinity stress triggers extensive transcriptional reprogramming, activating stress-responsive genes and signaling pathways, with transcription factors such as NAC, WRKY, MYB, and bZIP playing key roles [14,15]. Technological advances have improved our understanding of the molecular mechanisms involved in plant salinity stress responses, particularly the importance of hormone signaling (like abscisic acid and auxin) and the role of small RNAs (miRNAs) [16,17,18,19]. Genome editing technologies like CRISPR/Cas9 provide opportunities for precision modification of genes involved in salt stress responses, potentially leading to the development of salt-tolerant crop varieties [20,21].

However, the complex nature of salt tolerance traits, often involving multiple genes and regulatory networks, presents challenges [22,23]. Several research areas remain, including understanding the difference between plant responses to acute and chronic salinity stress, the role of the plant microbiome in modulating salinity stress responses, and the potential of nanotechnology [24]. With the deterioration of soil salinization caused by climate change, it is essential to adopt an integrative approach that combines physiological, molecular, and ecological perspectives in order to devise sustainable strategies for improving plant salt tolerance. This approach should encompass major crop species as well as the exploration of halophytes and wild relatives for unique salt tolerance traits [25]. Therefore, comprehending how plants react to salinity stress continues to be a crucial issue in the fields of plant biology and agriculture. Despite the significant progress made in unraveling the molecular and physiological mechanisms of salt stress adaptation, there are still many unanswered questions. The RNA-seq method has been utilized to analyze the salinity response mechanisms in species of the *Brassicaceae* family. Studies have shown that transcription factors and transporter genes play crucial roles in the salinity response process in *B. napus* and *B. juncea* [26,27]. Additionally, carbohydrate metabolism, hormone, and MAPK signaling pathways also play important roles in the salinity response process in *B. napus* [28]. Moreover, salinity stress significantly disrupts photosynthesis and the phenylpropanoid biosynthesis pathway in *B. napus* [29]. However, there is relatively little research on using RNA-seq to analyze the molecular mechanisms underlying salt stress response in *B. rapa* L. ssp. *Pekinensis*.

During production, it was observed that the carotenoid content in the tissues of the orange-heading Chinese cabbage ‘15 za-22’ significantly decreased when grown in high-salt soil. To systematically analyze the molecular mechanisms underlying salt stress response in the orange-heading Chinese cabbage ‘15 za-22’, a variety with high carotenoid content, plants were treated with 150 mM and 300 mM NaCl, then subjected to transcriptome sequencing analysis. Subsequently, the differentially expressed genes (DEGs) in plants treated with 150 mM and 300 mM NaCl were determined in comparison to the control group. We further analyzed the involvement of DEGs in both Gene Ontology (GO) and Kyoto Encyclopedia of Genes and Genomes (KEGG) pathways. Moreover, we employed weighted correlation network analysis (WGCNA) to pinpoint hub genes that are sensitive to salt stress. Our research provides data support to enhance our understanding of how *B. rapa* L. ssp. *Pekinensis* responds to salt stress, paving the way for futural gene functional studies.

## 2. Results

### 2.1. Salinity Stress Causes Chlorosis and Decreased Contents of Chlorophyll and Carotenoids in the Leaves of B. rapa L. ssp. Pekinensis

**Figure 1:**
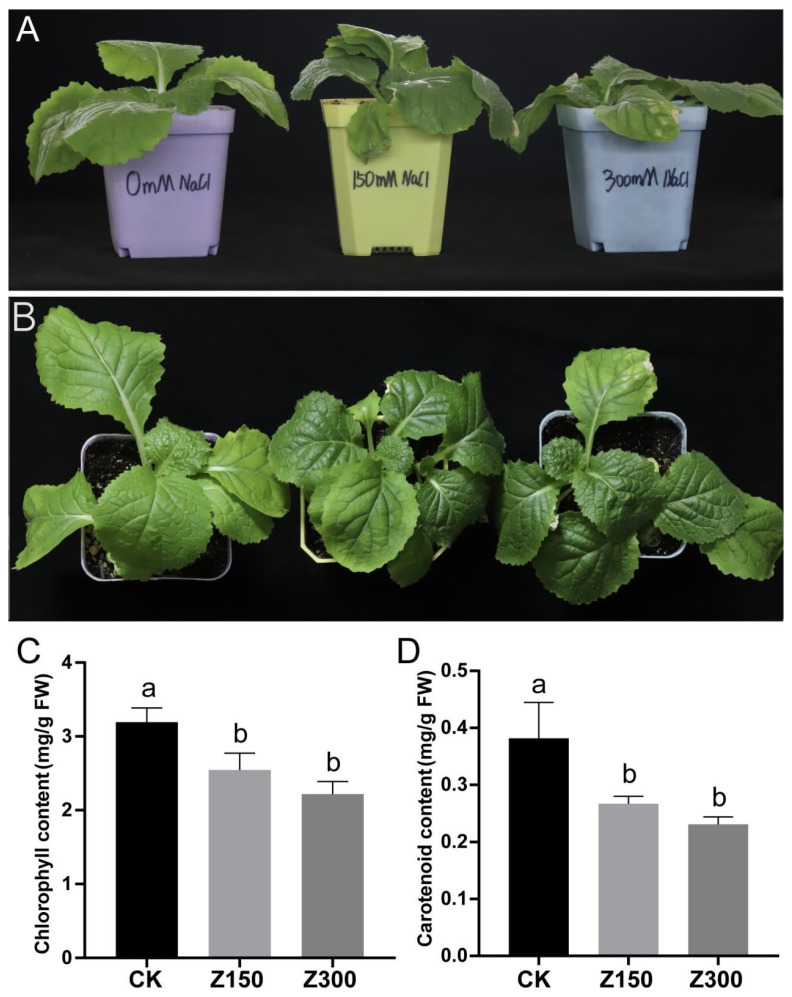
NaCl treatments cause chlorosis and decreased contents of chlorophyll and carotenoids in the leaves of *B. rapa* L. ssp. *Pekinensis*. Phenotypes of *B. rapa* L. ssp. *Pekinensis* from (**A**) side view shot and (**B**) aerial shot. Contents of (**C**) chlorophyll and (**D**) carotenoids in the leaves of *B. rapa* L. ssp. *Pekinensis*. CK: Hoagland’s nutrient solution. Z150: Hoagland’s nutrient solution + 150 mM NaCl. Z300: Hoagland’s nutrient solution + 300 mM NaCl. The letters on the bar chart represent different levels based on Duncan’s test, with a *p*-value of ≤0.05 as the threshold.

Salinity stress is one of the most important abiotic stresses in the environment that affects the normal growth and development of plants [30]. To understand the effects of salinity stress on the growth of *B. rapa* L. ssp. *Pekinensis*, we subjected *B. rapa* L. ssp. *Pekinensis* seedlings to different concentrations of NaCl treatment. The results showed that salinity stress significantly affected the growth of *B. rapa* L. ssp. *Pekinensis* seedlings (Figure 1). After NaCl treatment, the leaves of *B. rapa* L. ssp. *Pekinensis* seedlings exhibited slight wilting and noticeable chlorosis (Figure 1A,B). The chlorophyll and carotenoid contents in the leaves of *B. rapa* L. ssp. *Pekinensis* seedlings were significantly reduced relative to the control after NaCl treatment (Figure 1C,D).

### 2.2. Salinity Stress Triggers Reprogramming of Gene Expression

**Figure 2:**
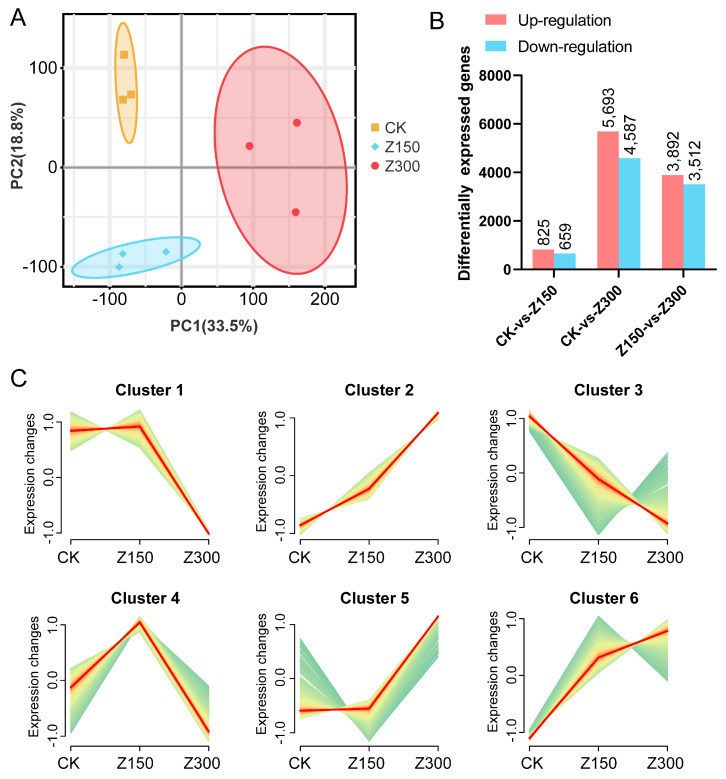
RNA-seq analysis of the leaves of *B. rapa* L. ssp. *Pekinensis* treated with different contents of NaCl. (**A**) Principal-component analysis. (**B**) Identification of differentially expressed genes (DEGs) among the experimental groups. (**C**) Expression trends of DEGs among the experimental groups. CK: Hoagland’s nutrient solution. Z150: Hoagland’s nutrient solution + 150 mM NaCl. Z300: Hoagland’s nutrient solution + 300 mM NaCl.

To investigate the impact of salinity stress on transcriptional regulation in *B. rapa* L. ssp. *Pekinensis*, we conducted RNA-seq analysis on the leaves of CK, Z150, and Z300 in *B. rapa* L. ssp. *Pekinensis* seedlings, with three biological replicates for each experimental group. The results of PCA (principal-component analysis) indicated good biological reproducibility among different experimental groups (Figure 2A). The analysis of DEGs revealed that compared to CK, Z150 had 825 up-regulated genes and 659 down-regulated genes, while Z300 had 5693 up-regulated genes and 4587 down-regulated genes. Additionally, Z300 had 3892 up-regulated genes and 3512 down-regulated genes compared to Z150 (Figure 2B). The gene set including all DEGs obtained from pairwise comparisons of all samples could be classified into six clusters based on their expression trends. In Cluster 1, the gene set showed no significant change in Z150 but a significant decrease in Z300. In Cluster 2, the gene set exhibited a sustained increase in expression in both Z150 and Z300. Cluster 3 showed a continuous decrease in expression in both Z150 and Z300. In Cluster 4, the gene set displayed an initial increase in Z150 followed by a decrease in Z300. Cluster 5 showed no significant change in Z150 but a significant increase in Z300, while Cluster 6 showed a significant increase in Z150 and a slight increase in Z300 (Figure 2C).

### 2.3. GO Enrichment Analysis of DEGs with Specific Expression Trends

**Figure 3:**
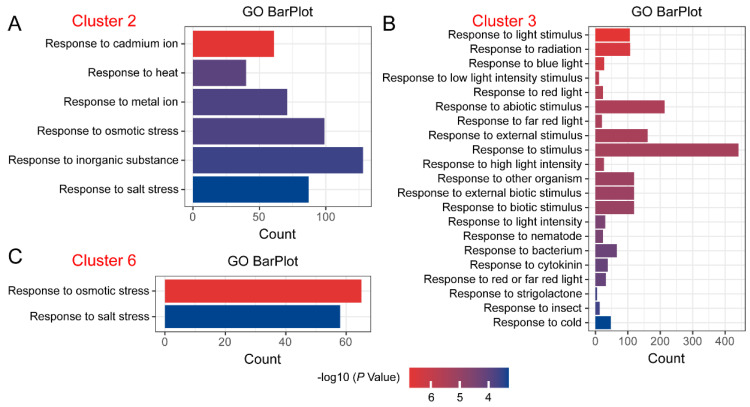
GO enrichment analysis of DEGs with specific expression trends. GO enrichment analysis of DEGs in (**A**) Cluster 2, (**B**) Cluster 3, and (**C**) Cluster 6.

Through clustering analysis of DEGs, we found that different DEGs exhibited distinct expression trends. We focused on DEGs that showed sustained up-regulation, such as those in Cluster 2 and Cluster 6, as well as those with sustained down-regulation, such as those in Cluster 3. The Gene Ontology (GO) enrichment analysis of DEGs in Cluster 2 revealed GO terms related to ‘response’ biological processes, including “Response to cadmium ion”, “Response to heat”, “Response to metal ion”, “Response to osmotic stress”, “Response to inorganic substance”, and “Response to salt stress” (Figure 3A). For DEGs in Cluster 3, the GO enrichment analysis showed numerous GO terms associated with ‘response’ biological processes, such as “Response to light stimulus”, “Response to abiotic stimulus”, “Response to biotic stimulus”, and “Response to cold” (Figure 3B). In Cluster 6, the GO enrichment analysis of DEGs identified two GO terms related to ‘response’ biological processes, namely “Response to osmotic stress” and “Response to salt stress” (Figure 3C).

### 2.4. DEGs’ Response to Salt Stress

**Figure 4:**
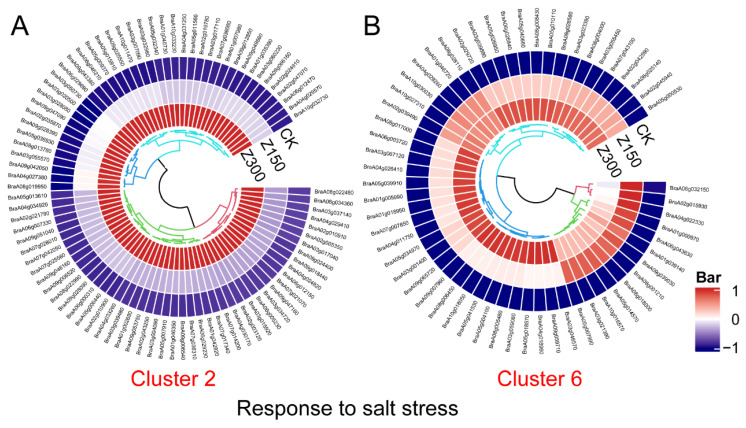
Expression profiles of DEGs annotated as “Response to salt stress” in Clusters 2 and 6. (**A**) DEGs in Cluster 2. (**B**) DEGs in Cluster 6. CK: Hoagland’s nutrient solution. Z150: Hoagland’s nutrient solution + 150 mM NaCl. Z300: Hoagland’s nutrient solution + 300 mM NaCl. Expression values were normalized using z-scores.

It is interesting that the Gene Ontology (GO) enrichment analysis of differentially expressed genes (DEGs) in both Cluster 2 and Cluster 6 enriched the “Response to salt stress” GO term. We identified these DEGs in Clusters 2 and 6. Expression analysis indicated that these DEGs were significantly induced during NaCl treatment (Figure 4). However, DEGs in Cluster 2 were slightly induced in Z150 but significantly induced in Z300 (Figure 4A), whereas DEGs in Cluster 6 were significantly induced in both Z150 and Z300 (Figure 4B). Gene functional annotation revealed that DEGs in Cluster 2 included heat shock proteins 70s (BraA02g003120 and BraA03g004090), peroxidases (BraA06g005230, BraA08g034360, and BraA09g066160), CBL-interacting protein kinase (BraA05g032340), glutathione S-transferases (BraA06g007330 and BraA07g036060), mitogen-activated protein kinase (BraA09g028390), P5CSs (BraA04g030170 and BraA05g006440), sodium hydrogen (BraA09g024400), sodium ion transport (BraA03g039480), and transcription factors (BraA02g019780, BraA03g020500, BraA03g050730, BraA03g055570, BraA04g029410, BraA07g017340, BraA07g028010, BraA08g019950, and BraA09g043380) (Figure 4A). DEGs in Cluster 6 included E3 ubiquitin–protein ligases (BraA05g040660 and BraA07g007850), mitogen-activated protein kinases (BraA03g023390 and BraA05g004100), P5CS (BraA03g021390), transcription factors (BraA04g018960 and BraA05g014570), and vesicle-associated membrane proteins (BraA01g005990, BraA08g018000, and BraA10g027310) (Figure 4B).

### 2.5. Salinity Stress Inhibits Photosynthesis and Carbon Metabolism

**Figure 5:**
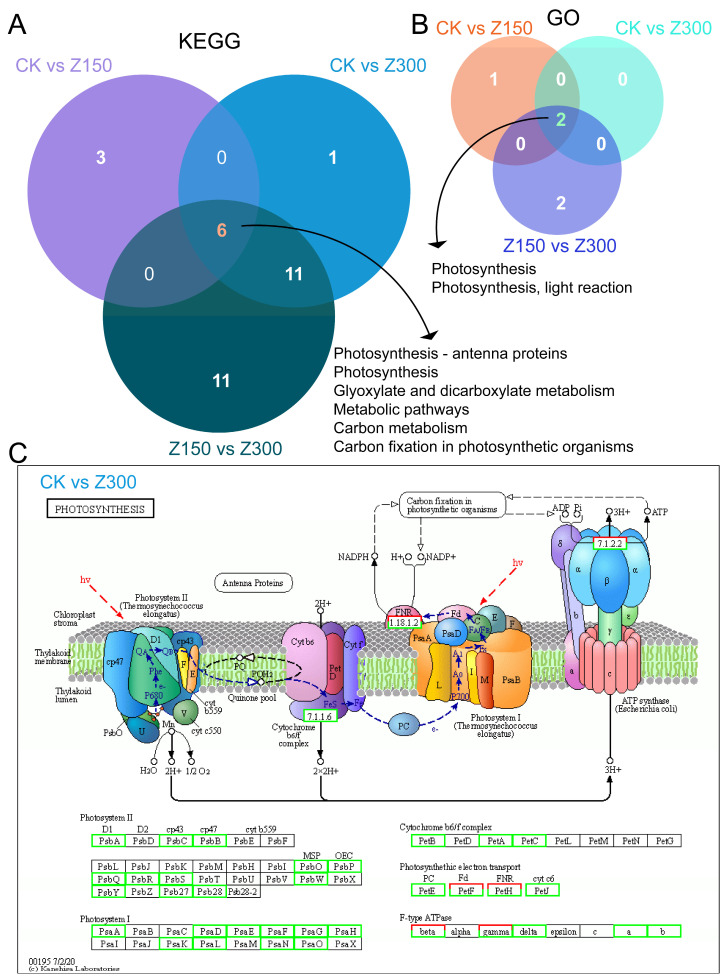
Salinity stress inhibits photosynthesis and carbon metabolism. (**A**) Common enriched KEGG pathways of DEGs among different experimental groups. (**B**) Common enriched GO terms of DEGs among different experimental groups. (**C**) Most DEGs in photosynthesis pathways were down-regulated between CK and Z300. CK: Hoagland’s nutrient solution. Z150: Hoagland’s nutrient solution + 150 mM NaCl. Z300: Hoagland’s nutrient solution + 300 mM NaCl. Genes with green borders represent down-regulated genes, genes with red borders represent up-regulated genes, genes with both green and red borders represent gene families with both down-regulated and up-regulated genes, and genes with black borders represent genes with no differential expression.

Although we have identified several important DEGs related to salinity stress through the analysis of DEGs with specific expression trends, we are still interested in understanding the overall impact of NaCl treatment on the biological processes of *B. rapa* L. ssp. *Pekinensis*. Previously, we identified up-regulated and down-regulated genes among different experimental groups and conducted GO and KEGG enrichment analyses on these DEGs (Figure 2B). The GO enrichment analysis of DEGs between CK and Z150, DEGs between CK and Z300, and DEGs between Z150 and Z300 revealed significant effects on photosynthesis (Figures S1–S3). Similarly, the KEGG enrichment analysis of these DEGs also showed significant impacts on photosynthesis and carbon metabolism (Figures S4–S6). Venn analysis indicated six commonly enriched KEGG pathways, including “Photosynthesis-antenna proteins”, “Photosynthesis”, “Glyoxylate and dicarboxylate metabolism”, “Metabolic pathways”, “Carbon metabolism”, and “Carbon fixation in photosynthetic organisms” (Figure 5A). Additionally, two common enriched GO terms were identified, namely “Photosynthesis” and “Photosynthesis, light reaction” (Figure 5B). These results strongly suggest that the biological process of photosynthesis was disrupted during salinity stress. Taking DEGs between CK and Z300 as an example, most of the DEGs in the photosynthesis pathway were down-regulated in response to NaCl treatment (Figure 5C). This result is consistent with the MapMan analysis (Figure S7). Additionally, we also observed differences in the biological processes and KEGG pathways involved in DEGs between Z300 and Z150 samples compared to CK. For example, compared to CK, DEGs in Z150 were significantly enriched in the biological process of “photosynthesis, light harvesting in photosystem I”, while DEGs in Z300 did not show this enrichment (Figures S1 and S2). Furthermore, DEGs in Z150, when compared to CK, were enriched in KEGG pathways such as “MAPK signaling pathway—plant”, “Glycine, serine and threonine metabolism”, and “Peroxisome”, whereas DEGs in Z300, when compared to CK, were enriched in the “Citrate cycle (TCA cycle)” (Figures S4 and S5).

### 2.6. WGCNA Identifies Hub Genes Involved in Salinity Stress Response

**Figure 6:**
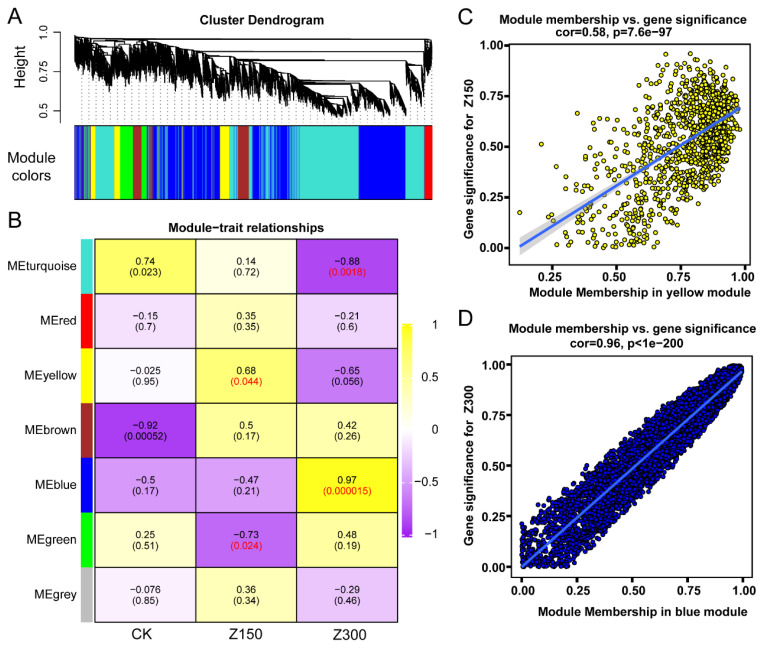
WGCNA of hub genes in salinity stress response in *B. rapa* L. ssp. *Pekinensis.* (**A**) Cluster of expressed genes. (**B**) Relationships of gene modules with different experimental groups. (**C**) Yellow gene module. (**D**) Blue gene module. CK: Hoagland’s nutrient solution. Z150: Hoagland’s nutrient solution + 150 mM NaCl. Z300: Hoagland’s nutrient solution + 300 mM NaCl.

WGCNA is an important tool for identifying hub genes in RNA-seq analysis. In order to discover hub genes involved in the response to salinity stress, we used the WGCNA package to cluster the expressed genes into different gene modules (Figure 6A). Subsequently, we performed an association analysis between the gene modules and different experimental groups, where the yellow module was significantly positively correlated with Z150, and the blue module was significantly positively correlated with Z300 (Figure 6B). Further analysis revealed a correlation coefficient of 0.58 with a *p*-value of 7.6 × 10^−97^ between “Module Membership in yellow module” and “gene significance for Z150” (Figure 6C) and a correlation coefficient of 0.96 with a *p*-value of less than 1.0 × 10^−200^ between “Module Membership in blue module” and “gene significance for Z300” (Figure 6D).

**Figure 7:**
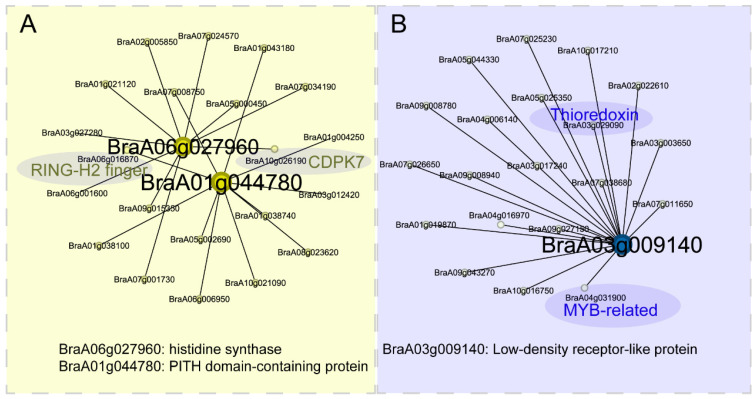
Co-expression networks of (**A**) yellow gene module and (**B**) blue gene module.

Then, we constructed co-expression networks for both the yellow and blue gene modules. For the yellow gene module, the co-expression networks revealed two hub genes, namely *BraA06g027960* (histidine synthase) and *BraA01g044780* (PITH domain-containing protein). Among the nodes connected to these hub genes, we identified two interesting genes, *BraA10g026190* (CDPK7) and *BraA06g016870* (RING-H2 finger) (Figure 7A). For the blue gene module, the co-expression networks identified one hub gene, *BraA03g009140* (low-density receptor-like protein). Among the nodes connected to this hub gene, we found two interesting genes, *BraA04g031900* (MYB-related) and *BraA03g029090* (Thioredoxin) (Figure 7B).

### 2.7. Salinity Stress Inhibits Carotenoid Metabolism

**Figure 8:**
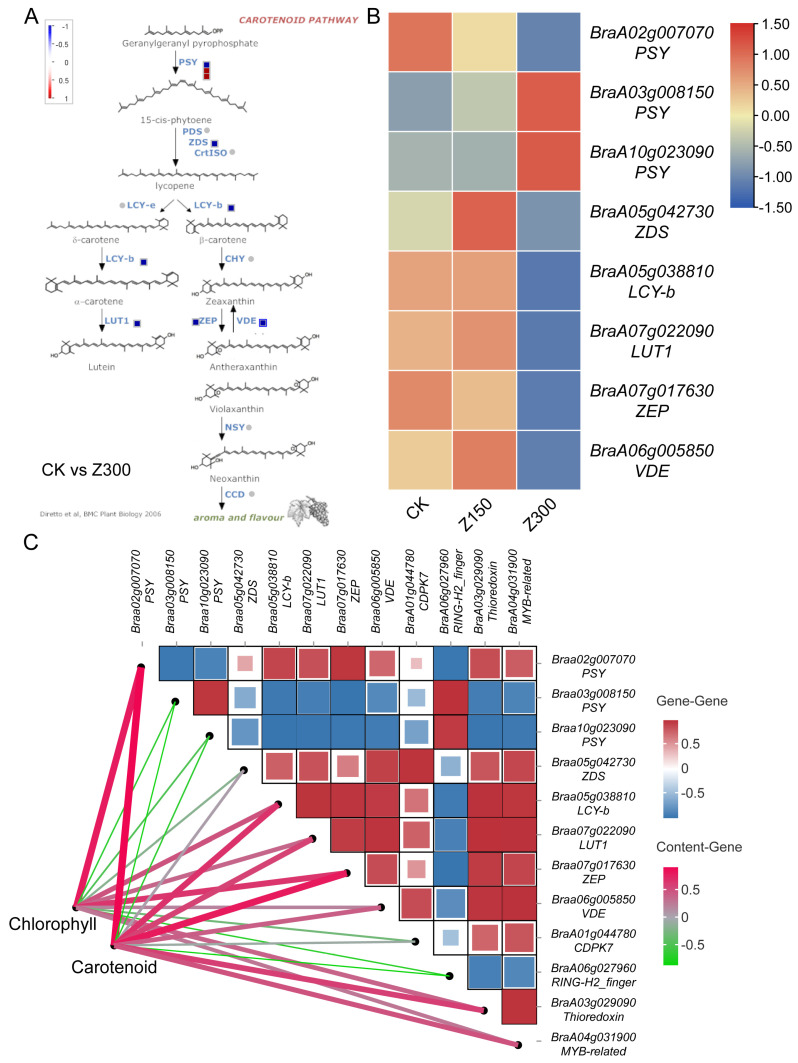
DEGs involved in the carotenoid pathway between CK and Z300. (**A**) Metabolic pathways of carotenoid metabolism in plants [31]. (**B**) Expression profiles of DEGs involved in carotenoid metabolism of *B. rapa* L. ssp. *Pekinensis*. (**C**) The correlation between hub genes and carotenoid-metabolism-related genes and carotenoid contents. CK: Hoagland’s nutrient solution. Z150: Hoagland’s nutrient solution + 150 mM NaCl. Z300: Hoagland’s nutrient solution + 300 mM NaCl. Expression values were normalized using z-scores.

Physiological index measurements revealed a significant decrease in carotenoid content in *B. rapa* L. ssp. *Pekinensis* leaves. Analyzing the genes in the carotenoid pathway, nine differentially expressed genes (DEGs) were found between the CK and the Z300 treatment group (Figure 8A). These DEGs included three phytoene synthase (PSY) genes, one zeta-carotene desaturase (ZDS) gene, one lycopene β-cyclase (LCY-b) gene, one lutein epoxidase (LUT1) gene, one zeaxanthin epoxidase (ZEP) gene, and one violaxanthin de-epoxidase (VDE) gene (Figure 8B). Among the three *PSY* genes, one was down-regulated and two were up-regulated, while the remaining six genes were all down-regulated (Figure 8B). To further clarify the relationship between hub genes generated from WGCNA and carotenoid metabolism and chlorophyll content, we analyzed the expression levels of DEGs in the carotenoid pathway with hub gene expression levels, as well as the correlation with carotenoid and chlorophyll content. We found that *BraA10g026190* (*CDPK7*), *BraA04g031900* (*MYB*-related), and *BraA03g029090* (*Thioredoxin*) were positively correlated with most of the DEGs in the carotenoid pathway, including *BraA02g007070* (*PSY*), while they were negatively correlated with *BraA03g008150* (*PSY*) and *BraA10g023090* (*PSY*). Conversely, *BraA06g016870* (*RING*-*H2 finger*) was negatively correlated with most of the DEGs in the carotenoid pathway but positively correlated with *BraA03g008150* (*PSY*) and *BraA10g023090* (*PSY*). Interestingly, *BraA04g031900* (*MYB*-*related*) and *BraA03g029090* (*Thioredoxin*) were positively correlated with carotenoid and chlorophyll content, while *BraA06g016870* (*RING*-*H2 finger*) was negatively correlated with carotenoid and chlorophyll content. *BraA10g026190* (*CDPK7*) showed no significant correlation with carotenoid and chlorophyll content (Figure 8C).

**Figure 9:**
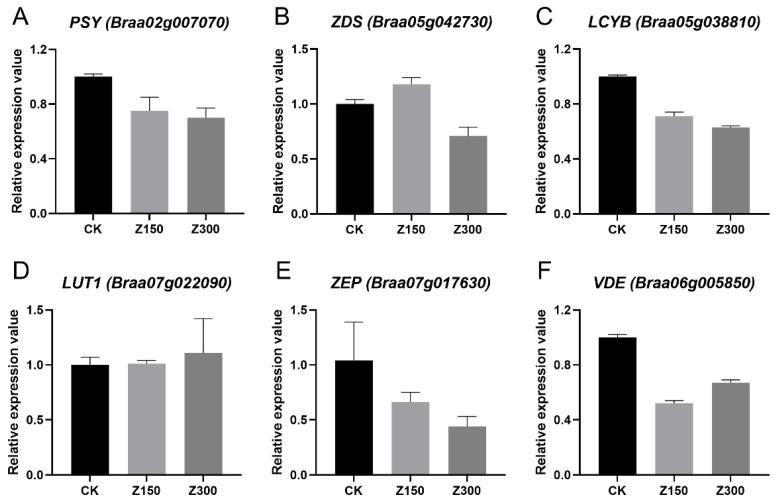
RT-qPCR analysis of DEGs in the carotenoid pathway. (**A**) PSY (*Braa02g007070*). (**B**) ZDS (*Braa05g042730*). (**C**) LCYB (*Braa05g038810*). (**D**) LUT1 (*Braa07g022090*). (**E**) ZEP (*Braa07g017630*). (**F**) VDE (*Braa06g005850*). CK: Hoagland’s nutrient solution. Z150: Hoagland’s nutrient solution + 150 mM NaCl. Z300: Hoagland’s nutrient solution + 300 mM NaCl.

RT-qPCR was performed to validate the reliability of the RNA-seq data. We selected six DEGs from the carotenoid pathway for analysis, and the results showed that the RT-qPCR results were consistent with the RNA-seq analysis results (Figure 9). This indicates that the RNA-seq data are reliable and further suggests that the results of the previous analysis are also reliable.

## 3. Discussion

The present study provides comprehensive insights into the physiological and molecular responses of *B. rapa* L. ssp. *Pekinensis* to salinity stress, highlighting the detrimental effects on growth, pigment degradation, and photosynthetic efficiency, as well as the complex transcriptional reprogramming that occurs under stress conditions.

**Salinity stress decreases plants chlorophyll and carotenoid contents**

Salinity stress represents a major environmental challenge that profoundly impacts plant growth and development through a cascade of physiological and molecular disruptions [32]. For instance, chlorophyll contents showed decreased in wheat during salt stress [33]. The chlorophyll and flavonoid content in the leaves of pepper significantly reduced during treatment with NaCl [34]. In this study, we observed that *B. rapa* L. ssp. *Pekinensis* seedlings exposed to NaCl treatment exhibited visible wilting and chlorosis. These symptoms align with the well-established effects of salinity stress, which often leads to a decrease in chlorophyll [35]. The significant decline in chlorophyll levels highlights the detrimental impact of salinity on the plant’s photosynthetic machinery [36]. A decrease in chlorophyll levels may be due to two reasons: inhibited chlorophyll synthesis or enhanced chlorophyll degradation [35]. In our study, further research is needed to investigate the reasons for the decrease in chlorophyll content, in order to understand the molecular mechanism by which salt stress affects leaf chlorophyll content.

A previous study has shown that relatively low-content NaCl treatment can increase the carotenoid content in tomato fruits [37]. Another study revealed that 100 mM NaCl treatment significantly increased the carotenoid content, while 150 mM NaCl treatment significantly decreased the carotenoid content in *Solanum nigrum* [38]. In buckwheat (*Fagopyrum esculentum* M.), 50 and 100 mM NaCl treatments significantly increased the carotenoid content in the sprouts [39]. Therefore, in response to salt stress, moderate salt stress can increase the carotenoid content in plants, while higher-intensity salt stress may decrease the carotenoid content [37,38,39]. Accordingly, increasing the total carotenoids in plant tissues will enhance the plant’s tolerance to salt stress [40]. In our study, we observed a significant decrease in carotenoid content in the leaves of *B. rapa* L. ssp. *Pekinensis* under NaCl treatments, indicating that salinity stress has severely disrupted the metabolic homeostasis of *B. rapa* L. ssp. *Pekinensis*. Chlorophyll degradation is a hallmark response to abiotic stress, indicative of damage to the photosynthetic apparatus, while the reduction in carotenoids, which are critical for photoprotection and oxidative stress mitigation, further compromises the plant’s resilience [41]. Collectively, these findings suggest that salinity stress not only disrupts photosynthesis but also diminishes the plant’s capacity to manage oxidative stress, thereby exacerbating the overall physiological damage.

**Salinity stress triggers reprogramming of transcriptional regulation in plants**

In response to high salt concentrations, plants activate complex molecular mechanisms to adapt and survive [42]. Several families of TFs have been identified as central players in transcriptional reprogramming under salinity stress. For example, the ethylene response factor (ERF) family plays a pivotal role in mediating salinity stress responses. OsERF106MZ in rice enhances salinity tolerance by alleviating ABA-mediated root growth inhibition [43]. Salinity tolerance in wheat is enhanced by TaERF3, leading to significant increases in proline and chlorophyll levels [44]. SlERF5 in tomato plants modulates water and proline content to enhance salinity tolerance [45]. Overexpression of *GmERF*3 in tobacco plants leads to improved tolerance to salt [46]. In this study, three *ERF* genes, namely *BraA03g055570*, *BraA07g028010*, and *BraA08g019950*, were significantly up-regulated during salinity stress. Moreover, MYB/MYC TFs are involved in the regulation of osmotic adjustment and antioxidant defense pathways. Transgenic rice plants with OsMYB6 and OsMYB91 show improved tolerance to salinity stress [47,48]. We also found four MYB genes, *BraA03g050730*, *BraA07g017340*, *BraA04g018960*, and *BraA09g043380*, induced by salinity stress. Additionally, WRKY TFs modulate the expression of genes involved in stress signaling and defense responses [49]. In our study, four WRKY genes, namely *BraA02g019780*, *BraA03g020500*, *BraA04g029410*, and *BraA05g014570*, showed significant up-regulation in response to NaCl treatments.

**Salinity stress triggers transcriptional regulation of carotenoid metabolism in *B. rapa***

The observed significant decrease in carotenoid content in *B. rapa* L. ssp. *Pekinensis* leaves under salinity treatment highlights the sensitivity of carotenoid metabolism to environmental or experimental perturbations. Carotenoids are critical for photoprotection, light harvesting, and antioxidant activity, and their depletion may reflect stress adaptation or metabolic disruption [50]. The identification of nine DEGs in the carotenoid pathway provides mechanistic insights into this phenomenon. Notably, the mixed regulation of three *PSY* genes (one down-regulated and two up-regulated) suggests a complex regulatory response. As PSY catalyzes the rate-limiting step in carotenoid biosynthesis [51], up-regulation of two *PSY* isoforms could indicate a compensatory mechanism to counteract the overall decline in carotenoid production. However, the down-regulation of downstream genes (*ZDS*, *LCY-b*, *LUT1*, *ZEP*, and *VDE*) likely creates bottlenecks in the pathway, leading to reduced flux and metabolite accumulation. For instance, reduced *LCY*-b expression could limit β-carotene synthesis [52], while down-regulated *VDE* might impair the xanthophyll cycle [53].

The integration of hub genes from WGCNA with carotenoid-related DEGs reveals potential regulatory nodes influencing this pathway. The positive correlation of *BraA04g031900* (*MYB*-*related*) and *BraA03g029090* (*Thioredoxin*) with most carotenoid DEGs and their positive association with carotenoid and chlorophyll content suggests these hub genes act as enhancers of carotenoid metabolism. MYB transcription factors are well-known regulators of secondary metabolism, including carotenoid biosynthesis [54], while thioredoxins modulate redox states of enzymes, potentially activating pathway components [55]. Conversely, *BraA06g016870* (*RING*-*H2 finger*), a negative regulator linked to ubiquitination, may suppress carotenoid synthesis by destabilizing key enzymes or transcription factors, supported by its negative correlation with most pathway DEGs and metabolite levels. Intriguingly, its positive association with two *PSY* genes (*BraA03g008150* and *BraA10g023090*) implies isoform-specific regulatory crosstalk, possibly fine-tuning spatial or temporal carotenoid production. The lack of correlation between *BraA10g026190* (*CDPK7*) and carotenoid/chlorophyll content suggests its role may lie outside direct regulation of this pathway, perhaps in calcium-mediated stress signaling that indirectly influences metabolic outputs [56].

These findings underscore the interplay between transcriptional regulators and metabolic flux. The coordinated down-regulation of carotenoid genes alongside chlorophyll decline aligns with the interdependence of these pigments in chloroplast function. The negative correlation of RING-H2 finger with both metabolites further implies a shared regulatory mechanism impacting plastid homeostasis. However, the study raises questions about the salinity treatment’s specific nature and its broader physiological implications. Future work could validate hub gene functions via knockout/overexpression studies and assess post-transcriptional modifications to clarify mechanisms. Additionally, exploring the spatial distribution of carotenoids and gene expression within leaf tissues could resolve discrepancies between transcript levels and metabolite accumulation. Overall, this integrative analysis advances our understanding of carotenoid regulation in *B. rapa* L. ssp. *Pekinensis* and identifies candidate genes for engineering stress-resilient or nutritionally enhanced crops.

**NaCl treatment disturbs plant development and immunity**

Salinity stress negatively affects various stages of plant development, from seed germination to reproductive growth. Salinity stress alters the balance of plant hormones, such as auxins, cytokinins, and gibberellins, which are critical for growth and development [57]. Moreover, salinity stress leads to a decrease in chlorophyll content in plant leaves [33,36], affecting photosynthesis and inhibiting the expression of light-response-related genes, thereby impacting plant growth and development. In this study, we found that many genes inhibited by salt stress are related to light response, indicating that the decreased chlorophyll content caused by salt stress has already significantly affected the metabolic processes of the photosynthetic pathway at the gene transcription level. Salinity stress not only weakens plant growth but also disturbs the plant’s immune system [58]. In this study, several down-regulated DEGs are involved in biotic stress response during NaCl treatments, particularly in plant immunity.

Additionally, we observed that treating *B. rapa* L. ssp. *Pekinensis* with different concentrations of NaCl significantly altered the gene expression pattern, suggesting that salinity stress dynamically reprograms transcriptional networks to modulate adaptive responses. The interplay between salinity-induced developmental impairments and compromised immunity underscores the multifaceted challenges faced by *B. rapa* L. ssp. *Pekinensis* under NaCl stress. The down-regulation of biotic-stress-responsive genes alongside photosynthetic and carotenoid pathway disruptions suggests a trade-off between abiotic stress adaptation and pathogen defense, potentially leaving plants vulnerable to concurrent biotic threats. This dual burden highlights the importance of hub genes like *MYB-related* and *Thioredoxin*, which may coordinate cross-stress resilience by balancing metabolic and defense outputs. The identification of *RING-H2 finger* as a negative regulator further emphasizes the need to fine-tune stress signaling networks. These insights position *B. rapa* L. ssp. *Pekinensis* as a model for dissecting stress crosstalk and inform strategies to enhance crop resilience through targeted genetic or agronomic interventions, particularly in saline-prone environments. Future studies should explore dynamic stress interactions and validate candidate genes in field conditions to bridge molecular findings with practical applications.

Overall, this study investigated the salt stress response in *B. rapa* L. ssp. *Pekinensis* and integrated transcriptional, metabolic, and physiological changes triggered by salinity stress. Sodium influx disrupts ion homeostasis, activating calcium signaling and MAPK cascades, which modulate transcriptional reprogramming. Key regulators include ERF, MYB, and WRKY transcription factors, which enhance osmolyte biosynthesis, antioxidant defense, and stress-responsive genes while suppressing growth-related pathways. Hub genes like MYB-related and Thioredoxin promote carotenoid synthesis and redox balance, whereas RING-H2 finger ubiquitinates positive regulators, suppressing carotenoid/chlorophyll synthesis. Salinity stress inhibits photosynthesis and carotenoid metabolism, leading to ROS accumulation, chlorosis, and growth arrest. Moderate stress allows partial adaptive responses, while severe stress overwhelms these mechanisms, causing metabolic collapse. This study identifies potential targets for engineering resilience, such as overexpressing MYB-related and Thioredoxin or knocking out RING-H2 finger, and provides a scaffold for validating gene functions and exploring stress-specific crosstalk using multi-omics approaches.

## 4. Materials and Methods

**Plant materials**

Orange-heading Chinese cabbage (*B. rapa* L. ssp. *Pekinensis*) ‘15 za-22’ seedlings were subjected to salt stress. After 30 days of seedling cultivation, they were divided into three experimental groups. The control group was irrigated with Hoagland’s nutrient solution, named the control group or CK. Experimental group 1 was irrigated with Hoagland’s nutrient solution + 150 mM NaCl, named Z150. Experimental group 2 was irrigated with Hoagland’s nutrient solution + 300 mM NaCl, named Z300. After treatment for three days, the 3rd–4th leaves from top to bottom were taken as experimental materials. They were collected and rapidly frozen with liquid nitrogen. A portion was used to determine chlorophyll and carotenoid content, while another portion was used for RNA-seq analysis. All experiments were conducted with three or more biological replicates.

**Chlorophyll and carotenoid content measurement**

The quantification of chlorophyll and carotenoid content was conducted following slightly modified, previously established methodologies [59]. After thoroughly grinding the leaves, they were soaked in 95% ethanol for 30 min until the tissue completely lost its green color. After centrifugation at 12,000 rpm for 5 min, the supernatant was collected and the volume adjusted. The absorbance of the extract was measured at 665 nm, 649 nm, and 470 nm. The chlorophyll content was calculated as 18.08 × A649 + 6.63 × A665, while the carotenoid content was calculated as 4.08 × A470 + 3.31 × A665 − 11.64 × A649.

**RNA extraction, RNA-seq library construction, and sequencing**

Total RNA was extracted from leaf samples using RNA Extraction Kits (Qiagen, Germany). Subsequently, mRNA with poly(A) tails was enriched by magnetic beads with Oligo(dT) and then fragmented using a buffer solution. The fragmented mRNA was used as a template to synthesize the first cDNA strand in the presence of random oligonucleotides as primers in the M-MuLV reverse transcriptase system (NEB, Beijing, China). Subsequently, the RNA strand was degraded by RNaseH (NEB, China), and the second cDNA strand was synthesized using dNTPs (NEB, China). The purified double-stranded cDNA was subjected to end repair, A-tailing, and sequencing adapter ligation. cDNA fragments of approximately 200 bp were selected using AMPure XP beads (NEB, Beijing, China), followed by PCR amplification and purification of the PCR products with AMPure XP beads to generate the final library. To ensure sequencing quality, stringent quality control measures were implemented during library construction, including the following analyses: agarose gel electrophoresis to assess RNA integrity and detect DNA contamination, a nanophotometer spectrophotometer to determine RNA purity, a Qubit2.0 Fluorometer for accurate RNA quantification, and an Agilent 2100 bioanalyzer for precise examination of RNA integrity. Sequencing was conducted on the Illumina Hiseq4000 platform.

**RNA-seq analysis and DEGs identification**

Quality control was performed on the raw reads obtained from sequencing using fastp to filter out low-quality data, resulting in clean reads [60]. The transcript abundance was quantified using Kallisto with B. rapa cv. Chiifu V4.1 as the reference genome from the BRAD database (http://brassicadb.cn/, accessed on 13 October 2024) [61]. Subsequently, DESeq2 was employed to analyze the differentially expressed genes (DEGs) between different experimental groups, with genes having an absolute log2(fold change) greater than 1 and a false-discovery rate less than or equal to 0.05 considered as DEGs [62].

**GO and KEGG enrichment analysis**

GO consists of three ontologies that describe the molecular function, cellular component, and biological process of genes [63]. Firstly, we mapped the DEGs to various terms in the GO database (http://www.geneontology.org/, accessed on 25 October 2024) and calculated the number of DEGs for each term to obtain a list of DEGs associated with a specific GO function. Subsequently, we applied hypergeometric testing using the gogseasenior tool (https://www.omicshare.com/, accessed on 29 October 2024) to identify significantly enriched GO terms in the DEGs [64]. Similarly, to further understand the biological functions of DEGs, KEGG pathway enrichment analysis was conducted using hypergeometric testing with the pathwaygseasenior tool (https://www.omicshare.com/, accessed on 29 October 2024) to identify significantly enriched pathways [64].

**WGCNA**

WGCNA-shinyApp (https://github.com/ShawnWx2019/WGCNA-shinyApp, accessed on 30 October 2024) was utilized for Weighted Gene Co-expression Network Analysis (WGCNA). The raw gene count values are normalized using the variance-stabilizing transformation method, followed by two rounds of gene set filtering. Firstly, genes with count values below 10 in 90% of the samples are excluded. Subsequently, the “median absolute deviation” method is used for further refined gene filtering. The normalized count values of the retained genes are used to determine the appropriate soft threshold power. Next, a module network is constructed with parameters set as “minimum module size = 30” and “module merge cut height = 0.25”. The correlation between modules and trait data is calculated, and significant “module–trait” associations are used to identify hub genes.

**Correlation analysis**

To analyze the correlation between hub genes and carotenoid-metabolism-related genes and carotenoid contents, we utilized the “Dynamic Network Heatmap” tool available at https://www.omicshare.com/ (Accessed on 8 February 2025).

**RT-qPCR analysis**

To validate the reliability of the RNA sequencing analysis, we performed real-time quantitative polymerase chain reaction (RT-qPCR) validation. Total RNA was extracted using the same method as the RNA sequencing analysis. Subsequently, the total RNA was reverse-transcribed into first-strand cDNA using HiScript IV All-in-One Ultra RT SuperMix for qPCR (Vazyme, Nanjing, China). Six differentially expressed genes (DEGs) were selected for RT-qPCR, and the specific primers are listed in Table S1. The qPCR experiments were conducted using the StepOnePlus™ Real-Time PCR System (ABI, Foster City, CA, USA), with *B. rapa* L. ssp. *Pekinensis* GAPDH gene as the reference gene [65]. As we focus on carotenoid metabolism pathways in *B. rapa* L. ssp. *Pekinensis*, we selected DEGs from the carotenoid metabolism pathway as candidate genes for RT-qPCR analysis. The relative expression levels of the selected genes were determined relative to the expression level in the control group (CK) using the 2^−ΔΔCT^ method [66].

**Statistical analysis**

Statistical analyses were conducted using SPSS v25.0. The Duncan test was used to determine the significance of differences between groups, with *p*-values < 0.05 considered statistically significant.

## 5. Conclusions

This study elucidates the multifaceted response of *B. rapa* L. ssp. *Pekinensis* to salinity stress, encompassing physiological changes, transcriptional reprogramming, and metabolic disruptions. The degradation of chlorophyll and carotenoids, inhibition of photosynthesis, and activation of stress-responsive genes collectively highlight the plant’s struggle to maintain homeostasis under adverse conditions. The identification of key DEGs and hub genes provides valuable targets for future research aimed at enhancing salinity tolerance in crops. These findings contribute to a deeper understanding of the molecular mechanisms underlying plant stress responses and offer potential strategies for improving crop resilience in saline environments.

## Data Availability

Data will be made available on request. The original RNA-seq data have been uploaded to the NCBI_SRA database (PRJNA1204385).

## Supplementary Materials

The following supporting information can be downloaded at: https://www.mdpi.com/article/10.3390/plants14040566/s1. Figure S1. GO enrichment analysis of DEGs between CK and Z150. Figure S2. GO enrichment analysis of DEGs between CK and Z300. Figure S3. GO enrichment analysis of DEGs between Z150 and Z300. Figure S4. KEGG enrichment analysis of DEGs between CK and Z150. Figure S5. KEGG enrichment analysis of DEGs between CK and Z300. Figure S6. KEGG enrichment analysis of DEGs between Z150 and Z300. Figure S7. MapMan analysis of DEGs between CK and Z300 involved in photosynthesis. Table S1. Primers used in this study.

## Abbreviations

The following abbreviations are used in this manuscript:

DEGs	Differentially expressed genes
GO	Gene Ontology
WGCNA	Weighted Gene Co-expression Network Analysis
KEGG	Kyoto Encyclopedia of Genes and Genomes
PCA	Principal-component analysis
HSPs	Heat shock proteins
PODs	Peroxidases
CIPKs	CBL-interacting protein kinases
GSTs	Glutathione S-transferases
MAPKs	Mitogen-activated protein kinases
