# Supplementary figures and images for: Transcriptomic Analysis Reveals That the Photosynthesis and Carotenoid Metabolism Pathway Is Involved in the Salinity Stress Response in Brassica rapa L. ssp. Pekinensis
Source: Plants (Basel). 2025 Feb 13;14(4):566. doi: 10.3390/plants14040566 (PMC11859510; doi:10.3390/plants14040566)

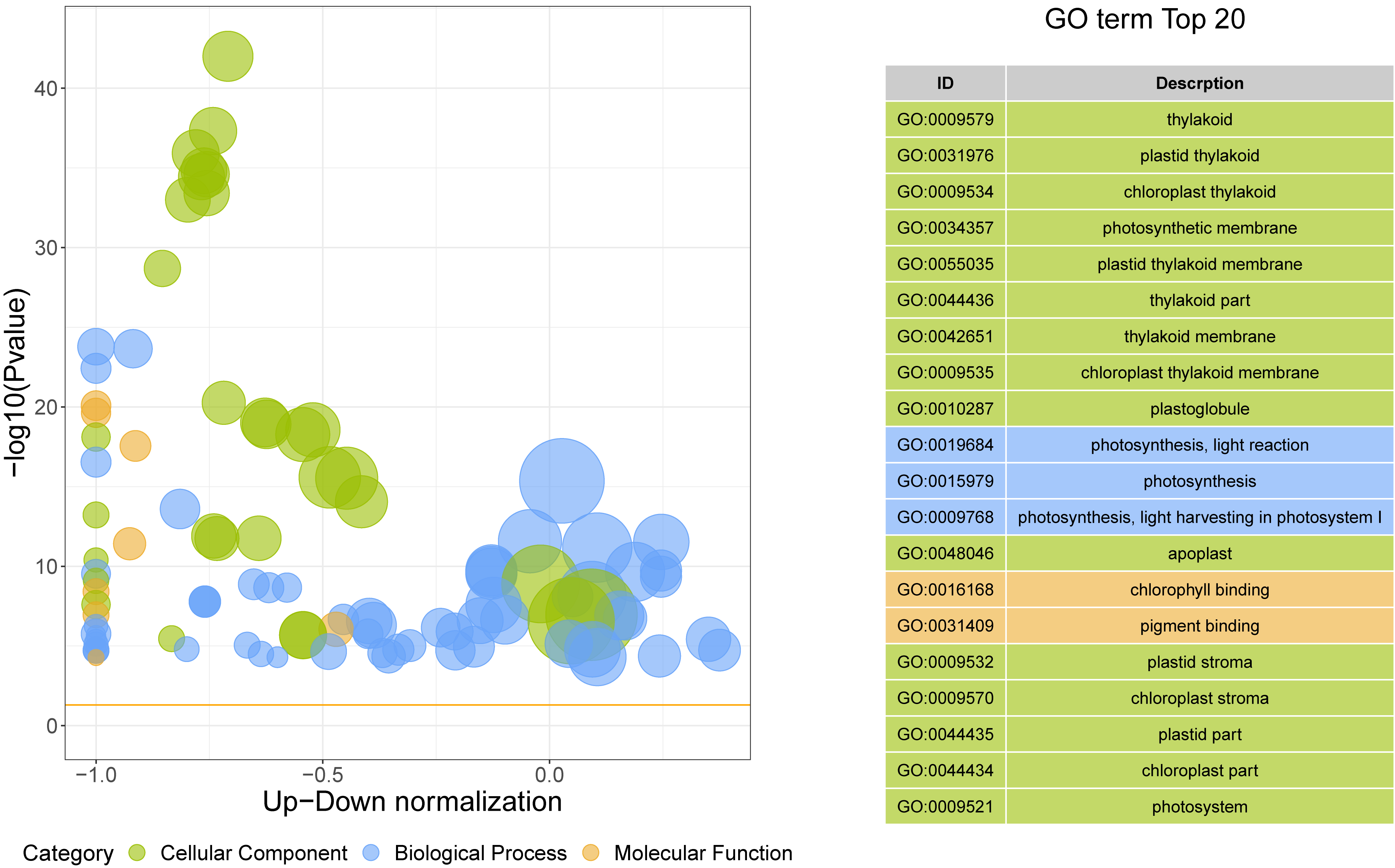

Supplement: Supplementary file 1 [file plants-14-00566-s001.zip › Figure S1.tif]

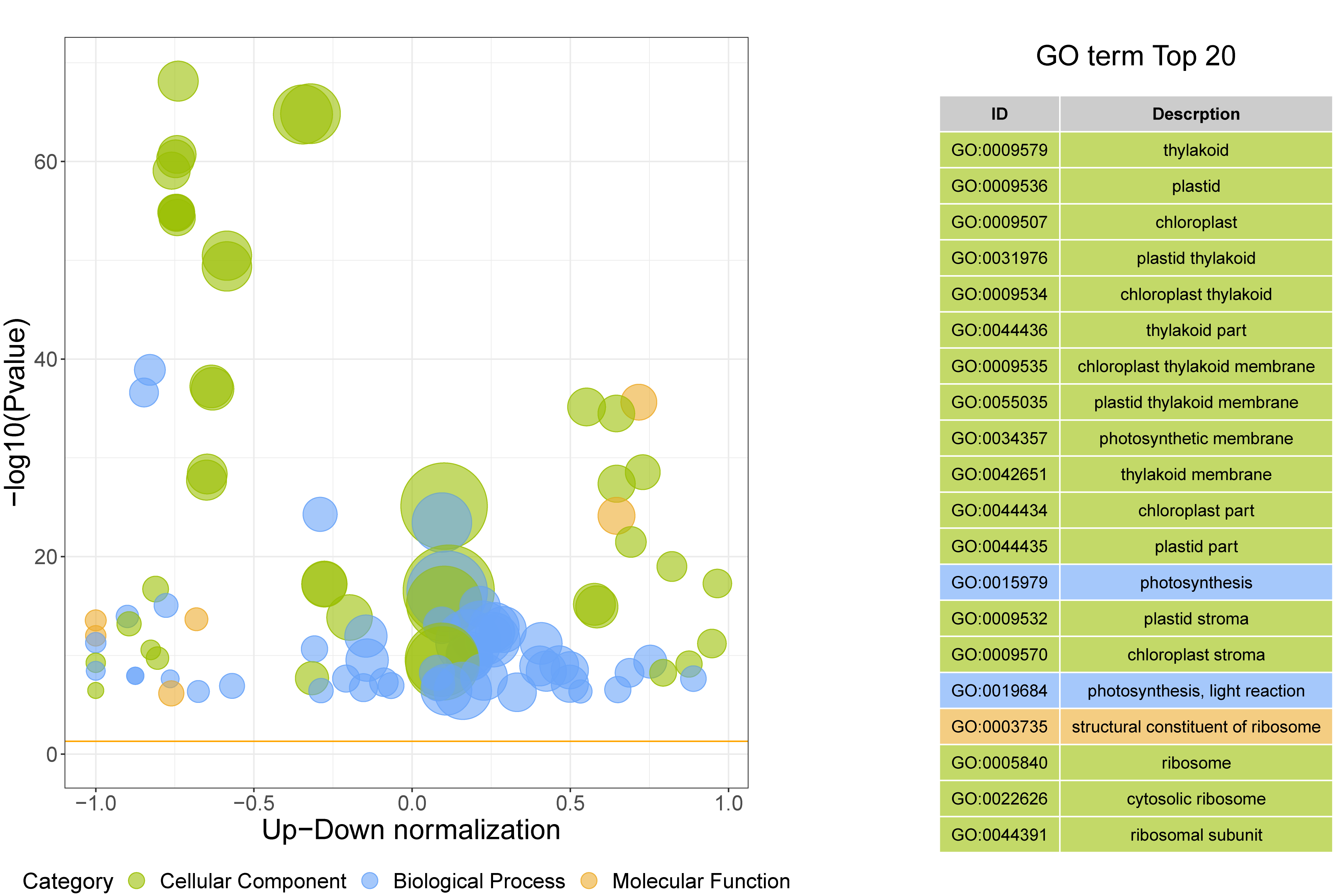

Supplement: Supplementary file 1 [file plants-14-00566-s001.zip › Figure S2.tif]

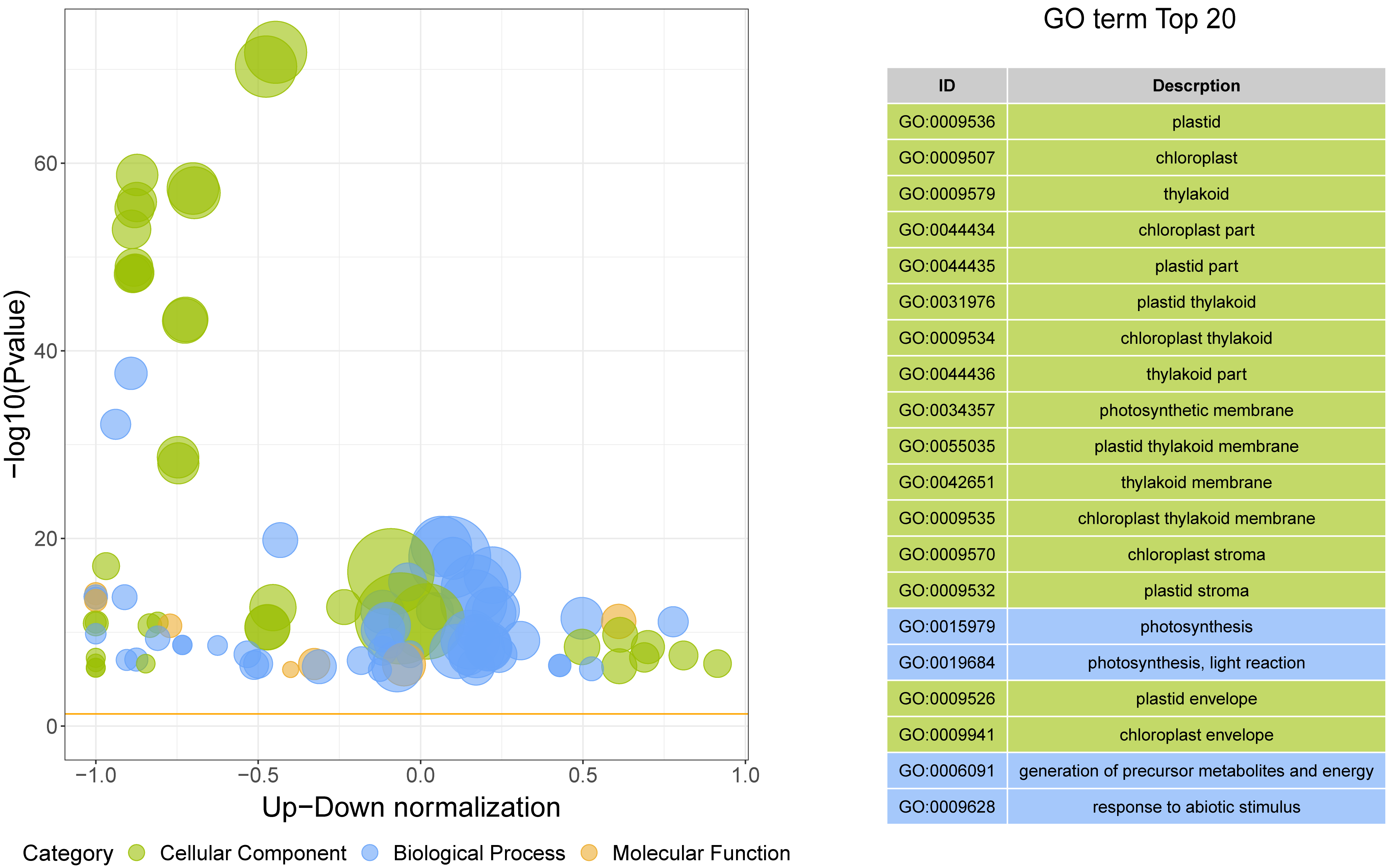

Supplement: Supplementary file 1 [file plants-14-00566-s001.zip › Figure S3.tif]

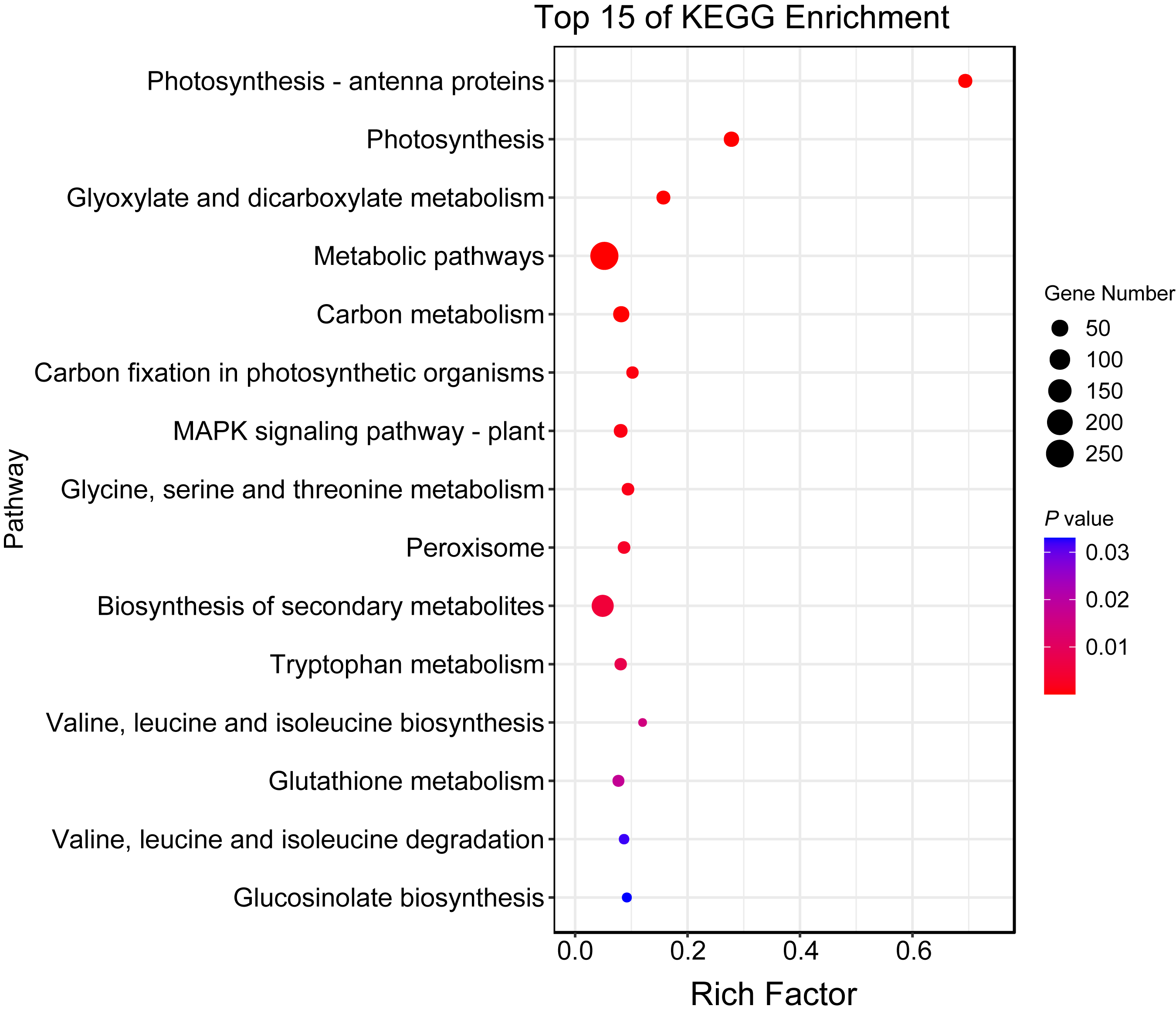

Supplement: Supplementary file 1 [file plants-14-00566-s001.zip › Figure S4.tif]

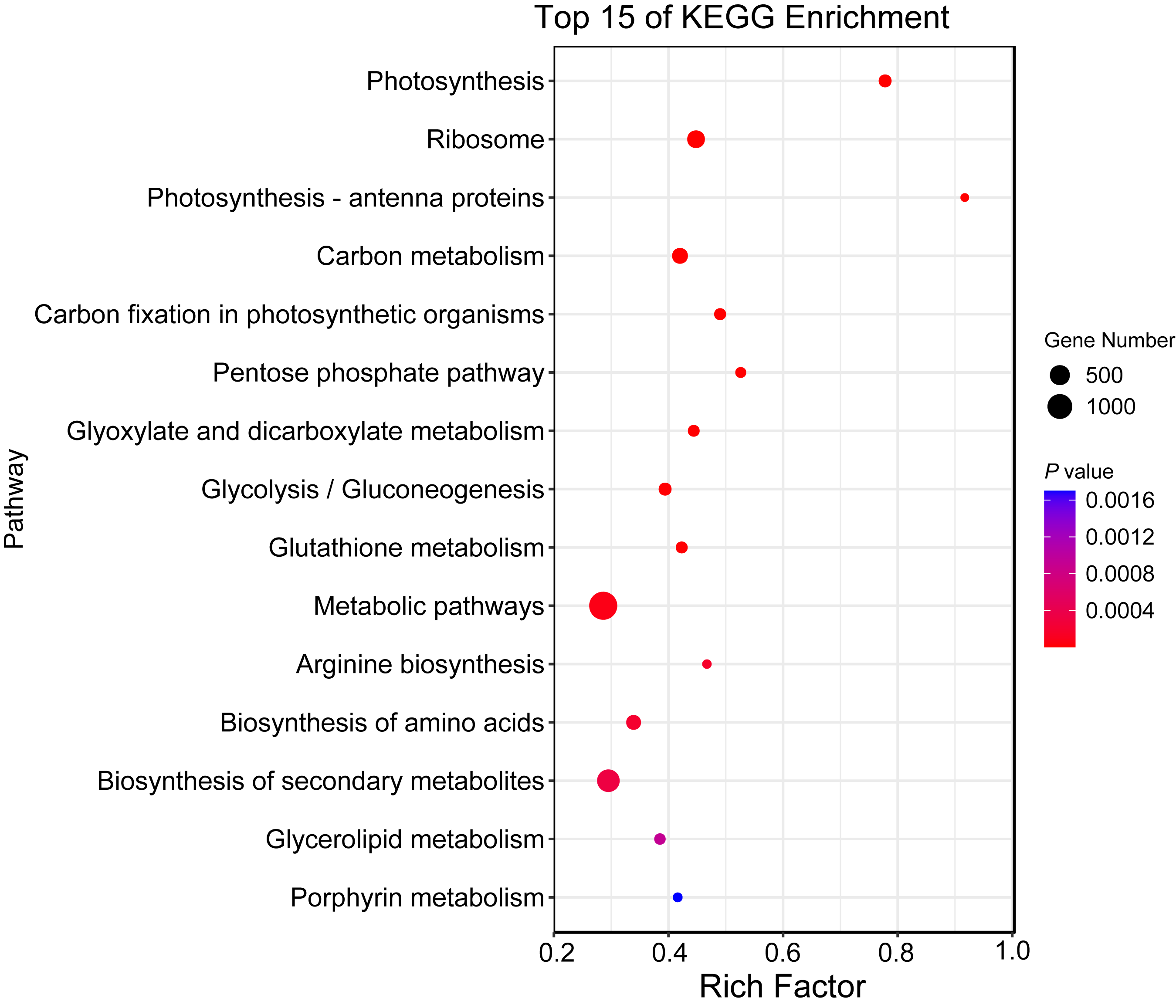

Supplement: Supplementary file 1 [file plants-14-00566-s001.zip › Figure S5.tif]

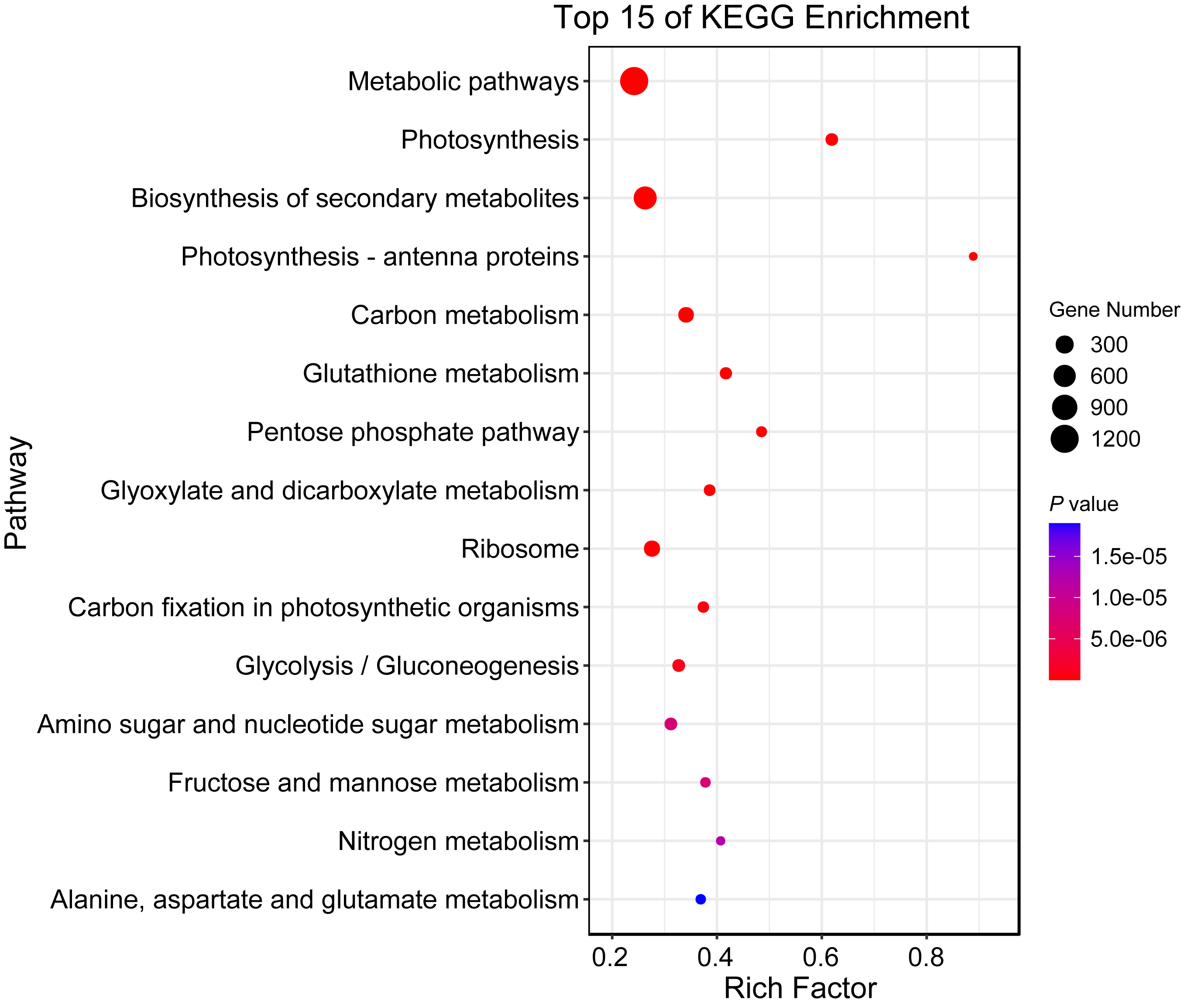

Supplement: Supplementary file 1 [file plants-14-00566-s001.zip › Figure S6.tif]

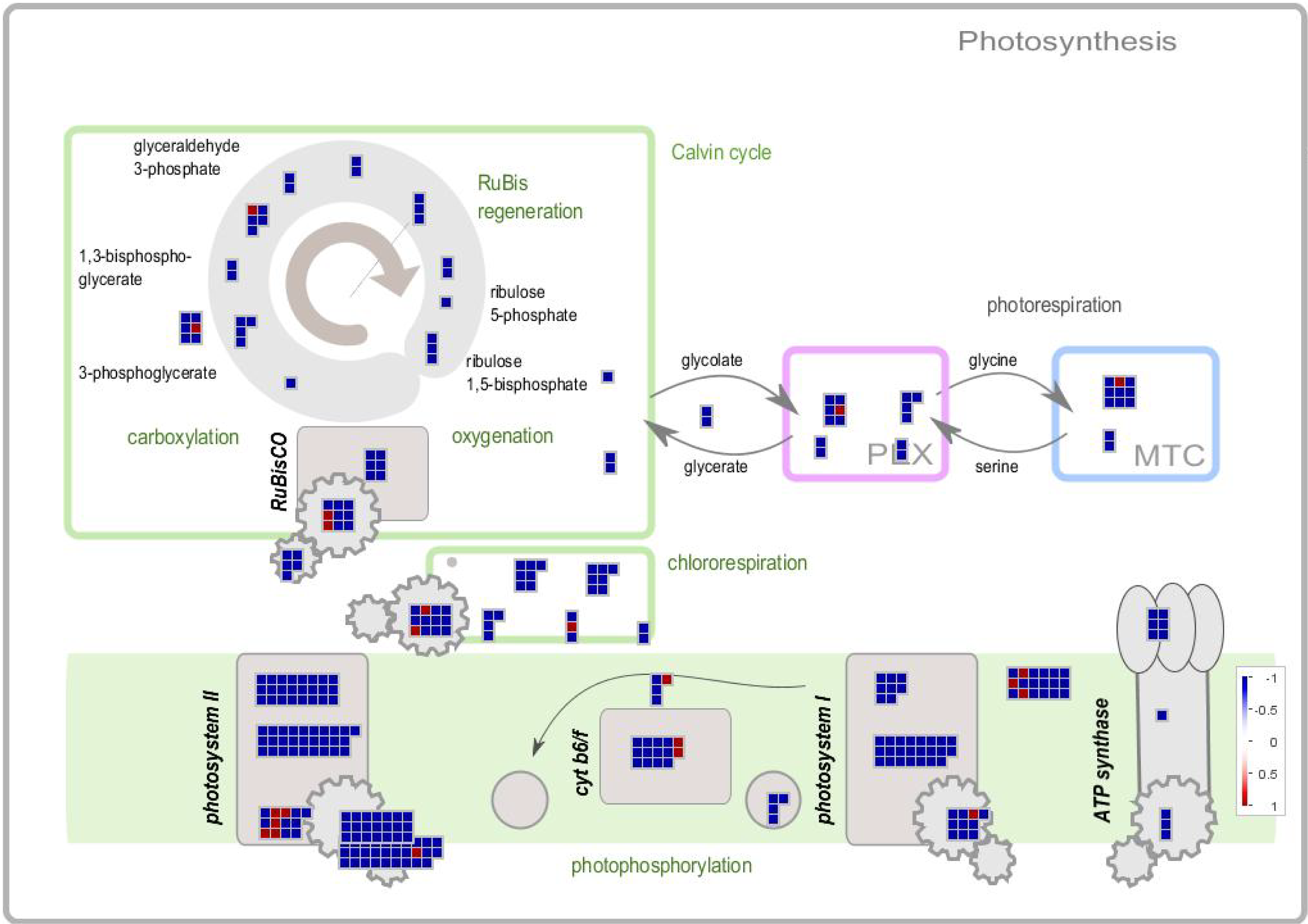

Supplement: Supplementary file 1 [file plants-14-00566-s001.zip › Figure S7.tif]
